# A frustratingly easy way of extracting political networks from text

**DOI:** 10.1371/journal.pone.0313149

**Published:** 2025-01-27

**Authors:** Naim Bro

**Affiliations:** 1 School of Government, Adolfo Ibanez University, Santiago, Chile; 2 Millennium Institute of Foundational Research on Data, Santiago, Chile; National Institute of Informatics, JAPAN

## Abstract

This study demonstrates the use of GPT-4 and variants, advanced language models readily accessible to many social scientists, in extracting political networks from text. This approach showcases the novel integration of GPT-4’s capabilities in entity recognition, relation extraction, entity linking, and sentiment analysis into a single cohesive process. Based on a corpus of 1009 Chilean political news articles, the study validates the graph extraction method using ‘legislative agreement’, i.e., the proportion of times two politicians vote the same way. It finds that sentiments identified by GPT-4 align with how frequently parliamentarians vote together in roll calls. Comprising two parts, the first involves a linear regression analysis indicating that negative relationships predicted by GPT-4 correspond with reduced legislative agreement between two parliamentarians. The second part employs node embeddings to analyze the impact of network distance, considering both with and without sentiment, on legislative agreements. This analysis reveals a notably stronger predictive power when sentiments are included. The findings underscore GPT-4’s versatility in political network analysis.

## Introduction

Identifying how political elites are related to each other is pivotal for understanding their influence on policy-making, governance, and the broader democratic process. Traditional methods for delineating these networks often rely on manual, labor-intensive analysis, which faces significant challenges in scalability and adaptability, particularly in the rapidly evolving landscape of political interactions.

Addressing this gap, this study introduces a novel application of GPT-4, a Large Language Model (LLM), in extracting political networks from textual sources. The novelty of this approach lies in the integration of GPT-4’s capabilities in entity recognition, relation extraction, entity linking, and sentiment analysis into a unified process. The case study focuses on a set of 1009 news clips that mention members of the Chilean Chamber of Deputies in the period November 2021 to September 2023. A critical aspect of this approach is the emphasis on sentiment analysis in the network extraction process. By analyzing the tone and context of interactions reported in the news, it differentiates between positive, negative, and neutral relationships. This sentiment-focused analysis offers a more refined and realistic representation of the political landscape, moving beyond mere co-occurrence or binary connections. This dimension is particularly important in a field that has been famously defined as the opposition between friends and enemies [1].

To empirically validate the extracted network, this study undertakes two experiments. The first employs regression analysis to assess the impact of having a connection reported in the media, as well as the sentiment (positive, negative, and neutral) associated to such connection, on the legislative agreement between parliamentarians. This analysis allows to quantify the relationship between the network connections identified by GPT-4 and actual legislative behavior. The second experiment extends this analysis by using node embeddings to measure the influence of network distance on legislative agreement.

The findings reveal a significant correlation between the sentiments identified by GPT-4 and the legislative agreement among parliamentarians. Negative sentiments predicted by the model are closely associated with decreased legislative agreement. Additionally, node embeddings showed that deputies closer in the network, especially in sentiment-weighted models, tend to exhibit higher legislative agreement, underscoring the predictive power of network distances in parliamentary voting patterns.

The contributions of this study are threefold. Firstly, it highlights the capacity of GPT-4, an accessible tool to many researchers in political science and sociology, to effectively discern entities and their interrelations within textual data. Secondly, it demonstrates that integrating sentiment analysis into political network construction significantly increases the real-world relevance of the extracted networks. Thirdly, it proposes the use of ‘legislative agreement’ [2]—the proportion of times in which two parliamentarians vote in the same way in roll calls—as a systematic metric for externally validating the networks extracted.

The paper is organized as follows: it begins with a review of existing literature and then outlines the data and methods used in this study. This is followed by a presentation of the results and concludes with a discussion and a conclusion.

## Related work

The extraction of political networks has garnered increasing attention, with data now spanning over 150 countries [3]. Traditional methods, such as those used by [4, 5], involve manual coding of relationships from various sources. While thorough, this approach is constrained by its labor-intensive nature and data availability. For instance, [4] manually coded biographical ties among Chinese political elites, finding that network centrality is associated with formal political influence. Similarly, [5] examined the Danish power elite by mapping their organizational membership, identifying a tightly connected group of 423 individuals who held significant sway over economic and political decisions. These studies highlight the depth of insights achievable through manual coding but also underscore the challenges in scalability.

Other studies, like those assessing Chinese Communist Party elites [6, 7], also rely on manually coded databases, limiting scalability.

A shift toward automation has been seen in approaches that scrape news outlets, employing Named Entity Recognition (NER) to identify political figures [8–11]. These methods enhance scalability but often overlook the qualitative nature of relationships, including their emotional valence. For instance, [8] defines actions associated with links but does not incorporate this information in the analysis.

Validation methods for extracted networks have also varied. Studies like [4, 12] examine the association between centrality measures and formal political influence, and [6] find the correlation between network distance and appointments to Leading Small Group within the Communist Party. These approaches make sense but are too specific to context to be applicable in other countries.

In contrast, this study employs GPT-4 for automated political network extraction, uniquely combining entity recognition, named entity linking, relation extraction, and sentiment analysis into a single process. This multi-purpose functionality allows for a more nuanced understanding of political networks, capturing not just the links but the nature and quality of these connections. Further, by introducing ‘legislative agreement’ as an external validation metric [2], this study provides a novel method to objectively assess the accuracy of the extracted networks. Legislative agreement quantifies the proportion of times two parliamentarians vote the same way, offering a direct measure of political alignment that is external to the dataset used for network extraction.

Related literature in computer science, particularly in joint entity and relation extraction (JERE), has seen significant advancements in recent years. For instance, several studies have presented models that integrate the BERT language model for improved feature representation and extraction accuracy [13–15]. These approaches have established new state-of-the-art benchmarks by effectively learning distinct contextual representations for entities and relations and addressing challenges such as overlapping triples and exposure bias [16, 17].

Other contributions include the development of tagging schemes that convert joint extraction tasks to more manageable forms [18, 19]. These methods have been shown to outperform existing pipelined and joint learning methods by leveraging advanced tagging and attention mechanisms.

While these approaches have made significant strides in the field, they often pose challenges in terms of accessibility in fields like political science and sociology. In contrast, GPT-4, as a tool readily accessible to a wide range of researchers, offers a more comprehensive and user-friendly approach to complex political network analysis. It allows users to perform text-to-graph operations without needing to delve into the technical complexities of deep learning models, thus democratizing access to advanced text analysis capabilities.

Moreover, by validating the extracted networks against legislative agreement data, this study bridges the gap between computational methods and real-world political behavior. This validation approach not only enhances the credibility of the extracted networks but also sets a precedent for future research to incorporate objective external metrics in network validation.

This paper leverages GPT-4’s capabilities to bridge the gap between advanced computational techniques and practical application in political network analysis, demonstrating that high-quality results can be achieved without the need for specialized technical expertise.

## Data and methods

### News dataset and graph extraction

The primary data source originates from Eventregistry, a service that aggregates news from various media outlets. The initial dataset included 42,385 Chilean news clips mentioned at least one member of the Chilean Chamber of Deputies between November 2021 and September 2023. Due to budget and time constraints, the analysis was conducted using a subset of news clips below the tenth percentile of word count, resulting in a reduced sample of 3,582 articles. As the focus of the study is the identification of relationships between pairs of parliamentarians, a final filter was applied to select only those news clips that mentioned at least two of the 155 deputies. This filtering process resulted in a final dataset of 1,009 news clips (for an example of news clip, refer to S6 Appendix).

While the dataset was filtered to include shorter news clips, this was a necessary step due to practical constraints such as budget and time limitations. The decision to limit the dataset by clip length introduces certain biases, particularly the exclusion of longer clips that might contain more complex political interactions, such as agreements or pacts. Nevertheless, the shorter clips offer valuable insights into media-reported political dynamics, as shorter media segments often focus on immediate and prominent interactions. This choice, while narrowing the data sample, still captures core relational dynamics reported in Chilean media during the selected period. Future research could address these limitations by including both short and long clips for a more comprehensive analysis.

GPT-4 was employed as an advanced text analysis tool to process this dataset, focusing on political news related to the Chilean Congress. The model’s capabilities in entity recognition, relation extraction, and sentiment analysis were leveraged to identify the relationships among members of the Chamber of Deputies. For sentiment analysis, GPT-4 has been shown to perform effectively in capturing the nuanced emotional tone in textual data, as demonstrated by recent studies [20]. Specifically, the model was prompted to cross-reference mentions in the news clips with a predefined list of deputies’ names, updating a structured dictionary that contained information about the nodes (representing individual deputies) and edges (representing the relationships between them). For details about the system and user prompts, see S1 Appendix.

The output consisted of 1,009 JSON-formatted strings, each representing a processed news article. Each string contained two key components: “nodes” representing the deputies and “edges” capturing the relationships and their associated sentiment scores between them. These JSON strings were subsequently converted into Python dictionaries and merged into a single consolidated dictionary by matching identical entities across the dataset.

This consolidated data was then transformed into a graph object using the NetworkX library in Python, resulting in a network that included 152 of the 155 deputies and represented 12,181 relationships between them. More formally:

The network *G* = (*V*, *E*) represents a directed graph, where:

- *V* is the set of vertices (nodes), representing deputies.
- *E* is the set of edges (links), representing relationships between deputies.

Each edge *e* has an associated sentiment *w*, defined as:

$$\begin{array}{c} w(e)=\text{Sentiment}\;\text{between}\;\text{the}\;\text{parliamentarians}\;\text{connected}\;\text{by}\;\text{edge}\;e \end{array}$$ (1)

Wherever multiple links connected a pair of deputies, these were consolidated in the following way. Initially, sentiments were mapped to numerical values: positive to + 1, negative to −1, and neutral to 0. These scores were then averaged to provide a consolidated sentiment score. Following this, the mean sentiment scores were reconverted into categorical sentiments using the following mapping: >0 = positive; 0 = neutral; <0 = negative.

**Fig 1:**
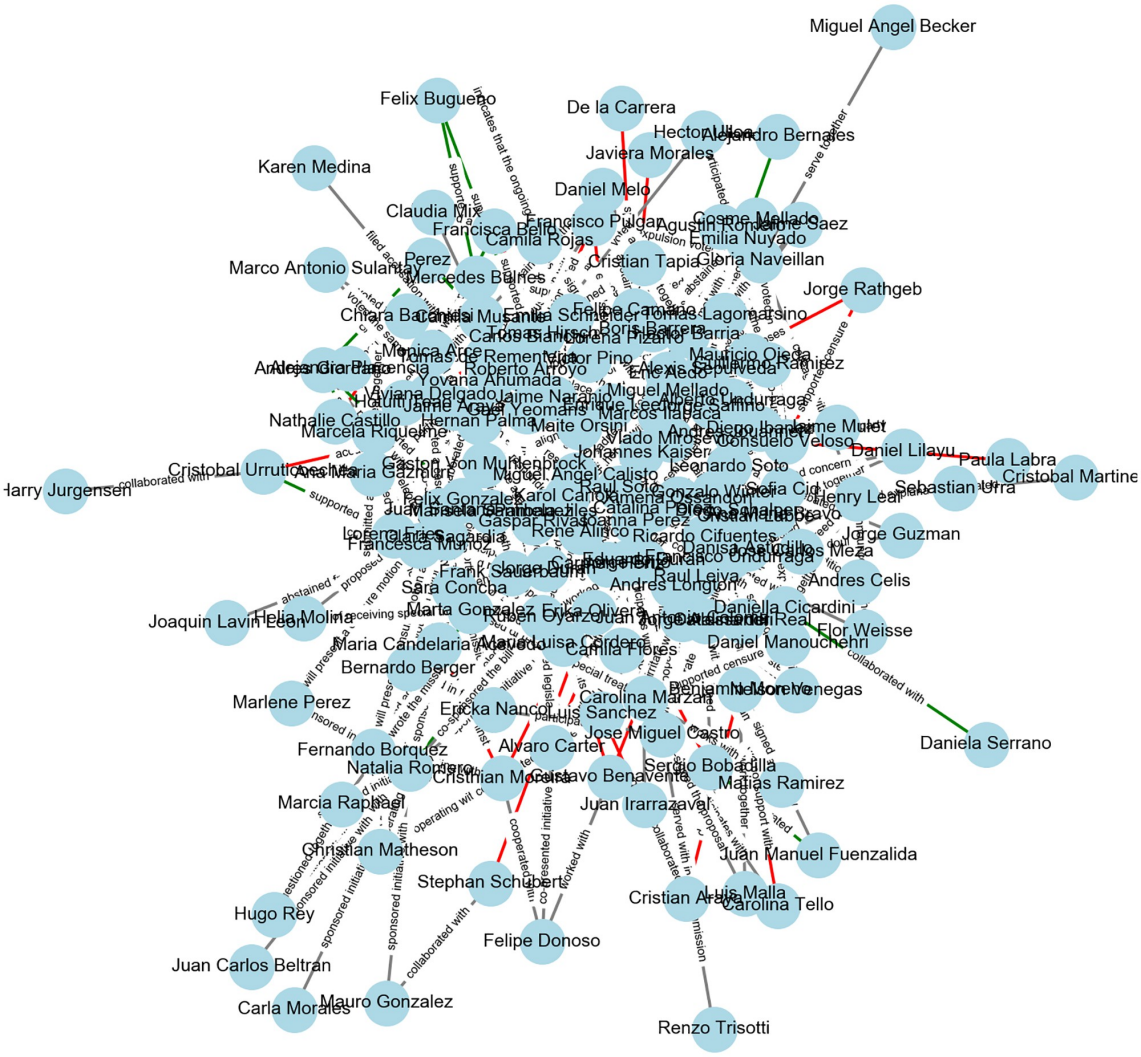
Network between Chilean deputies as extracted by GPT-4 from a news corpus (for a table with descriptive statistics of the network, refer to S4 Appendix).

Fig 1 presents the graph extracted from GPT-4, illustrating the complex web of relationships among Chilean deputies. The edges in the graph are labeled with the actions that connect two parliamentarians (e.g., “criticized”, “collaborated with”), and the colors of the edges represent the associated sentiments (green = positive, black = neutral, red = negative). This visual representation encapsulates the intricate political dynamics that were subsequently validated through empirical analysis.

### Empirical validation with legislative agreement

To empirically validate the network extracted through GPT-4, legislative agreement was utilized, i.e. the proportion of times in which two parliamentarians vote in the same way [2]. The legislative agreement metric serves as a direct and objective tool for this study as it quantitatively captures the degree of political alignment between parliamentarians.

The hypotheses tested in this study are as follows:

- H0: Deputies connected in the media-based network extracted by GPT-4 (whether positively or negatively) show no significant difference in legislative agreement compared to deputies neutrally connected.
- H1: Deputies positively connected in the media-based network extracted by GPT-4 are more likely to exhibit higher legislative agreement, while deputies negatively connected are more likely to exhibit lower legislative agreement, compared to those neutrally connected.

Legislative data was obtained from the official site of the Chilean Chamber of Deputies for the time window 11–03-2022 (when the current Congress started sessions) to 15–09-2023 (when the news corpus ends). For each potential pair of deputies, the proportion of legislation on which they voted the same way was calculated, relative to the total number of legislations in which they both participated. In this calculation, only the votes where both deputies actively participated (either voting “in favor” or “against”) were included, while abstentions or absences were excluded. Legislative agreement is more formally expressed as:

$$\begin{array}{c} \text{Leg.}\;{\text{Agree.}}_{ij}=\frac{\text{N}\;\text{of}\;\text{Same}\;\text{Votes}\;\text{between}\;\text{deputies}\;i\;\text{and}\;j}{\text{N}\;\text{of}\;\text{Joint}\;\text{Participations}\;\text{by}\;\text{deputies}\;i\;\text{and}\;j} \end{array}$$ (1)

While this approach provides a clear measure of explicit legislative alignment, it is important to recognize that abstentions and absences were not considered in the calculation. Abstentions, in particular, could hold significant informational value. As noted by [21], abstentions can often be a form of strategic behavior rather than indecision or neutrality. In some cases, deputies may abstain to avoid taking a contentious stance or to navigate complex political dynamics. Future studies could explore more nuanced measures that incorporate abstentions as an additional dimension of political behavior.

**Fig 2:**
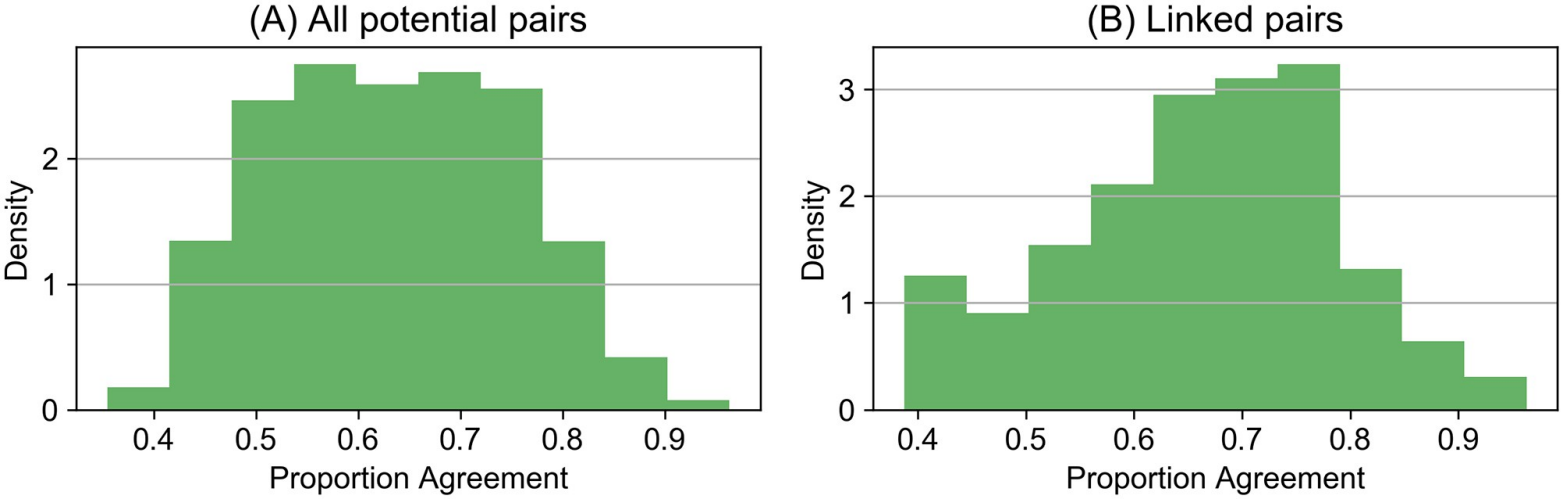


Fig 2 contains the density distribution of legislative agreement between all potential pairs of deputies (left panel) and between pairs of deputies connected in the corpus of news clips (right panel). As is visually apparent, the distribution resembles a normal distribution, especially when considering all potential pairs. In contrast, deputies connected in media clips are overrepresented in average and low levels of legislative agreement.

Subsequently, two experiments were conducted. The first consisted of two regressions to empirically validate the network extracted through GPT-4. The first regression assessed whether the presence of a link in the extracted graph (representing media co-occurrence) was predictive of higher legislative agreement levels. This analysis evaluates the validity of a media co-occurrence network, structured as follows:

$$\begin{array}{c} \text{Leg.}\;\text{Agree.}=\beta_{0}+\beta_{1}\times \text{Link}+\beta_{2}\times \text{Controls}+\varepsilon \end{array}$$ (2)

The second regression analysis examined the effect of sentiments (positive or negative, compared to neutral) identified by GPT-4 on legislative agreement. This approach provided a more nuanced quantitative assessment of the network extraction method’s validity. The regression model for this analysis is represented by:

$$\begin{array}{c} \text{Leg.}\;\text{Agree.}=\beta_{0}+\beta_{1}\times \text{Pos.}\;\text{Sentiment}+\beta_{2}\times \text{Neg.}\;\text{Sentiment}+\beta_{3}\times \text{Controls}+\varepsilon \end{array}$$ (3)

In both regression analyses, the legislative agreement between deputies is expressed in terms of standard deviations to standardize the scale across different pairs and facilitate a more uniform comparison.

In addition to the primary analysis using GPT-4, we conducted a supplementary validation using GPT-4o-mini, a smaller variant of the model. Despite the reduced model size, the results remain largely consistent with the main findings. Detailed results from GPT-4o-mini are presented in S5 Appendix.

### Node embeddings

In the second experiment, the outcome variable remained legislative agreement, with the main independent variable shifting to the distance between pairs of nodes in the network. Distances were calculated using node embeddings generated using node2vec [22], an algorithm that generates vector representations of nodes in a graph by simulating random walks. Node2vec is particularly well-suited for this study as it encodes both the network’s local and global structural information. Subsequently, cosine similarity was used to produce the final distance measure.

Node2vec employs multiple random walks from each node to construct embeddings. In this study, each node underwent 20 random walks (number of walks = 20), providing a balance between neighborhood exploration and computational efficiency. The choice of 20 walks per node ensures a comprehensive exploration of the network without excessively increasing computational cost. The walk length was set at 10 steps (walk length = 10), focusing on the immediate vicinity of each node without delving into distant and potentially less relevant network areas. A walk length of 10 was chosen to capture local neighborhood structures, which are crucial for understanding the immediate relationships and interactions between parliamentarians.

For the embeddings, two scenarios were considered: unweighted and weighted walks. Unweighted walks treated each step as having an equal chance of moving to any neighboring node, capturing the general structure of the network. In contrast, weighted walks incorporated sentiment, assigning higher probabilities to positively-linked neighbors (1/sum of weights), halved probabilities for neutral relations (0.5/sum of weights), and zero probability for negative links.

Following the walk generation, the word2vec model from the Gensim library in Python processed these sequences, treating nodes as words in sentences. Embeddings were configured with a size of 64 (embedding size = 64) to capture a diverse range of features within the network. An embedding size of 64 was selected to provide sufficient capacity to represent complex relationships while avoiding overfitting.

A context window of 10 (context window = 10) was used to consider a broad range of neighbouring nodes when learning each embedding. The context window of 10 ensures that the embeddings capture both direct and slightly indirect relationships, which is important for understanding the broader context of parliamentary interactions.

For the regression analysis, cosine distances between pairs of parliamentarians were calculated using both unweighted and weighted embeddings. This dual approach allowed for a comparative assessment of their predictive capabilities regarding legislative agreement. Formally:

$$\begin{array}{c} \text{Leg.}\;\text{Agree.}=\beta_{0}+\beta_{1}\times \text{Distance}+\beta_{2}\times \text{Controls}+\varepsilon \end{array}$$ (4)

In these regressions, both the legislative agreement and the distances between pairs of deputies were expressed in terms of standard deviations. This standardization of distances ensures comparability and consistency in measuring the network’s influence on legislative behavior. As in experiment 1, the models include control variables ‘Same Party’, ‘Same Sector’, and ‘Same Region.’

The hypotheses tested in this second experiment are as follows:

- H0: The network distance between deputies, as calculated using node embeddings (whether weighted or unweighted), has no effect on their legislative agreement.
- H1: Deputies who are closer in the network (as indicated by smaller distances in node embeddings) are more likely to exhibit higher legislative agreement, while deputies who are farther apart are more likely to exhibit lower legislative agreement.

As a robustness check, S5 Appendix shows the results of experiment 2 conducted with GPT-4o-mini.

## Results

This section reports the results of regression analyses to validate the graph by assessing how well the extracted graph predicts legislative agreement between pairs of deputies. The first test measures the effect of links and their sentimental valence, while the second test goes beyond links to measure the effect of network distances between pairs of nodes on their legislative agreement.

### Experiment 1

**Fig 3:**
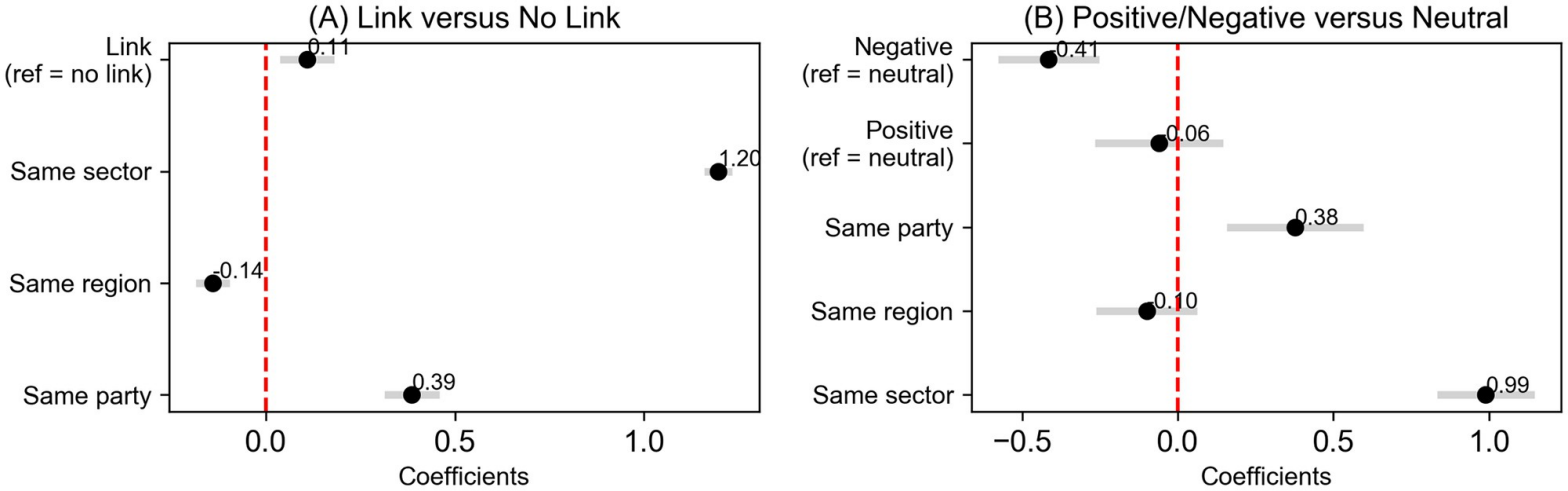
Coefficient predicting legislative agreement from the existence of a link (A), and the sentimental valence of links (B).

Fig 3 shows the results of the first set of regressions, measuring the effect of the co-occurrence of two politicians in the same news clip on their legislative agreement metric (left), and the effect of negative or positive links versus neutral links on legislative agreement (right) (for the full regression table, refer to S2 Appendix).

The analysis reveals some compelling findings that reinforce the efficacy of the proposed approach.

In Fig 3A, a link between two deputies is positively and significantly associated with legislative agreement, as indicated by a coefficient of *β* = 0.109***, expressed in standard deviations of the dependent variable. This means that moving from having no link to having a link increases the legislative agreement of two parliamentarians by 0.109 standard deviations. Given the statistical significance of this result (*p* < 0.001), we reject the null hypothesis (H0) and conclude that the existence of a link predicts higher legislative agreement.

In Fig 3B, negative sentiment as portrayed by the media is significantly associated with lower legislative agreement compared to neutral links, with a coefficient of *β* = −0.415***. Positive sentiment, however, does not have a statistically significant effect, with a coefficient of *β* = −0.060. As the negative sentiment effect is statistically significant (*p* < 0.001), we reject the null hypothesis (H0) and confirm that negative sentiment is associated with lower legislative agreement. However, since the positive sentiment effect is not statistically significant (*p* = 0.105), we fail to reject the null hypothesis regarding positive sentiment.

Same-party affiliation also shows a strong positive effect on legislative agreement, with a coefficient of *β* = 0.386*** in model (1) and *β* = 0.377*** in model (2), indicating that deputies from the same party tend to have much higher legislative agreement. Deputies representing the same region have a negative but small and only partly significant effect on legislative agreement, with *β* = −0.141*** in model (1) but an insignificant *β* = −0.100 in model (2). Finally, deputies within the same ideological sector show the strongest positive association with legislative agreement, with *β* = 1.197*** in model (1) and *β* = 0.989*** in model (2).

To ensure the robustness of these findings, we replicated the analysis using GPT-4o-mini. The results were consistent, with minor variations in the coefficients for shared party affiliation and sentiment polarity. The R-squared values from GPT-4o-mini were comparable to the larger model, indicating that even with a smaller model, the network’s predictive capacity remains strong. Full regression outputs for GPT-4o-mini are provided in S5 Appendix.

### Experiment 2

**Fig 4:**
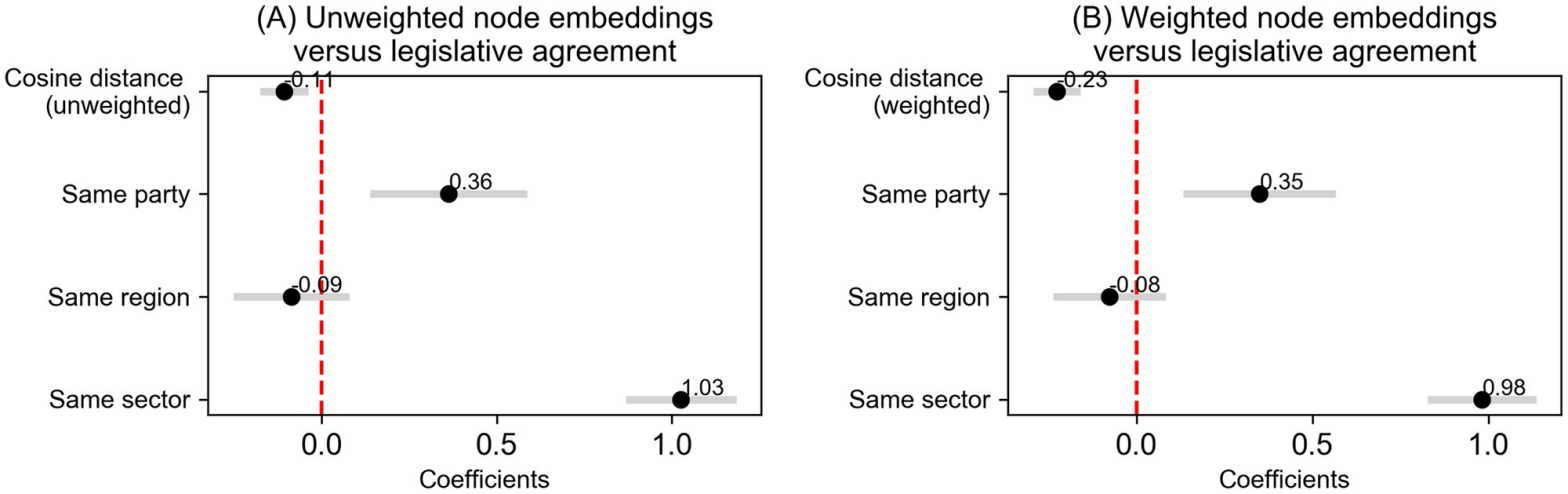
Coefficients from Regression Models predicting Legislative Agreement from Unweighted (A) and Weighted distance (B).

This phase of our study broadens the analytical scope established in Experiment 1, focusing on the network distance between nodes. We employed node2vec-generated embeddings to assess the impact of both weighted and unweighted cosine distances between pairs of deputies on their legislative alignment. The regression outcomes, depicted in Fig 4, offer insights into these dynamics (for the full regression table, refer to S3 Appendix).

In the analysis of the unweighted standard deviation of cosine distance, as illustrated in Fig 4A, we observe a significant negative coefficient of *β* = −0.116***, indicating that as the unweighted network distance between deputies increases, their likelihood of reaching legislative agreement decreases. This result, with statistical significance (*p* < 0.001), leads to the rejection of the null hypothesis (H0). We conclude that deputies who are closer in the network are more likely to vote similarly.

In Fig 4B, the weighted cosine distance shows an even stronger negative relationship, with a coefficient of *β* = −0.211***, indicating that the sentimental valence of ties amplifies the influence on legislative agreement. This result is statistically significant (*p* < 0.001), leading to the rejection of the null hypothesis (H0). Deputies with sentiment-weighted connections are shown to have a stronger likelihood of voting similarly, particularly when they are closer in the network.

The other variables included in the model (‘Same party’, ‘Same sector’, and ‘Same region’) behave in the same direction and with roughly the same magnitude as in Experiment 1. For example, deputies from the same party have a strong positive correlation with legislative agreement, with *β* = 0.383*** in model (1) and *β* = 0.354*** in model (2). The impact of representing the same sector is even stronger, with coefficients of *β* = 1.009*** and *β* = 0.987*** in models (1) and (2), respectively. Deputies from the same region, however, do not show a significant impact on legislative agreement, with coefficients close to zero in both models.

The results from the GPT-4o-mini embeddings corroborate those from GPT-4, with similar trends observed for both unweighted and sentiment-weighted distances. However, as expected with a smaller model, the effect sizes were slightly attenuated, particularly for weighted cosine distances. Despite this, the overall predictive power remains comparable. For a detailed comparison, please refer to S5 Appendix.

## Discussion

This study contributes significantly to the Joint Entity and Relation Extraction (JERE) literature by using GPT-4 for extracting political networks from text. The proposed methodology performs multiple functions simultaneously. Not only does it identify entities and their relationships, but it also discerns sentiments associated with these relationships and performs entity linking. For instance, it recognizes “presidente Boric” and “Gabriel Boric” as referring to the same individual when a closed list of reference names is provided. This multifaceted capability addresses a critical need in political network analysis.

An essential aspect of the proposed method’s utility lies in its use of tools familiar to political science and sociology practitioners. This approach lowers the barrier to entry for researchers and analysts who might not have specialized knowledge in specialized JERE methods. However, while the method shows promise, further research is necessary to compare its performance with alternative methodologies. Such comparative studies would help understanding the strengths and limitations of using GPT-4 in this context.

These findings have significant practical implications for policymakers and practitioners. For instance, sentiment-based link extraction can reveal emerging alliances or conflicts, providing early indicators of potential legislative gridlocks or successful policy initiatives. Moreover, understanding the network dynamics among legislators enables a deeper analysis of influence and power structures within the legislative body. Practitioners can identify key influencers and power brokers based on their centrality and sentiment-weighted relationships in the network. By continuously monitoring and analyzing political networks, policymakers can assess the impact of their initiatives in real time.

It is important to acknowledge the limitations introduced by the non-random filtering of the dataset, particularly the restriction to shorter news clips. While this decision was necessary due to practical constraints, it may exclude more complex interactions that could occur in longer clips, such as political agreements or in-depth discussions. As a result, the findings presented here are representative of the specific media-reported dynamics captured in shorter clips, but caution should be exercised in generalizing these results to broader political contexts. Future studies would benefit from including both short and long clips to provide a more comprehensive understanding of political relationships.

The quality of sentiment assignation by the proposed method is another critical discussion area. Visual examination suggests that while the classification of sentiments as negative is relatively accurate, the positive sentiment classification seems less precise. For instance, links associated with phrases such as “collaborated with”, “cooperating with”, and “co-signed initiative” were classified as neutral, whereas human judgement might want to classify them as positive. The model’s criteria for identifying positive sentiments may need adjustments to better align with human judgment. Improving the prompt used in the sentiment classification process could enhance the accuracy of this aspect, potentially by providing some examples from commonly occurring connective phrases.

In conclusion, the proposed method using GPT-4 offers a novel and valuable tool in political network analysis. Its ability to integrate entity identification, relationship extraction, sentiment analysis, and entity linking into a single process presents a significant advancement in this field.

## Conclusion

This study has demonstrated the potential of GPT-4 in extracting and analyzing political networks from text, specifically in the context of the Chilean Chamber of Deputies. The use of Large Language Models (LLMs) like GPT-4 has shown to be effective in not only identifying entities and their relationships but also in discerning the sentiments associated with these links. The approach presented in this paper bridges the gap between advanced computational techniques and their practical application in political science and sociology, making it accessible to researchers in these fields.

The validation of the method using “legislative agreement” as a metric confirms that the sentiments identified by GPT-4 correlate with the voting patterns of parliamentarians. The study reveals that negative sentiments, as identified by GPT-4, predict a decrease in legislative agreement, highlighting the model’s capability to capture the nuanced dynamics of political interactions.

Furthermore, the study introduces an efficient and scalable method for extracting political networks, which is significant considering the traditional methods’ limitations in scalability and adaptability. The ability of GPT-4 to perform joint entity and relation extraction, coupled with sentiment analysis, offers a comprehensive tool for political network analysis.

In conclusion, the use of GPT-4 in this study showcases a way for broader applications of LLMs in social sciences. The methodology proposed in this paper can be adapted and utilized in various political contexts, providing researchers with a powerful tool to analyze complex political landscapes. Future research should focus on expanding the dataset to include both short and long clips, allowing for a more robust and generalizable understanding of political relationships. Additionally, refining the sentiment analysis capabilities of the model and exploring its application in different political environments would harness its potential fully.

## Supporting information

S1 Appendix GPT-4 Prompt used to extract political networks from news. (PDF)

S2 Appendix Regression table for experiment 1. (PDF)

S3 Appendix Regression table for experiment 2. (PDF)

S4 Appendix Network descriptive statistics. (PDF)

S5 Appendix Experiments 1 and 2 with GPT-4o-mini. (PDF)

S6 Appendix News clip example, and resulting structured dictionary. (PDF)

## References

[1] Schmitt C. The Concept of the Political. University of Chicago Press; 1996.

[2] Henríquez PA, Sabat J, Sullivan JP Politicians’ Willingness to Agree: Evidence from the Interactions in Twitter of Chilean Deputies. Journal of Information Technology & Politics. 2023;20(1):92–111. doi: 10.1080/19331681.2022.2056278

[3] Commander SJ, Poupakis S. Political Networks Across the Globe. SSRN Electronic Journal. 2020. doi: 10.2139/ssrn.3568308

[4] Tokarev A, Margoev A, Prikhodchenko A. Exploring Biographical Ties among Party and State Leaders in China: A Social Network Analysis. Journal of Contemporary Asia. 2022;52(4):554–573. doi: 10.1080/00472336.2021.1954233

[5] Larsen AG, Ellersgaard CH. Identifying Power Elites—k-Cores in Heterogeneous Affiliation Networks. Social Networks. 2017;50:55–69. doi: 10.1016/j.socnet.2017.03.009

[6] Huhe N, Gallop M, Minhas S. Who Are in Charge, Who Do I Work With, and Who Are My Friends: A Latent Space Approach to Understanding Elite Coappearances in China. Social Networks. 2021;66:26–37. doi: 10.1016/j.socnet.2021.01.002

[7] Keller FB. Moving Beyond Factions: Using Social Network Analysis to Uncover Patronage Networks Among Chinese Elites. Journal of East Asian Studies. 2016;16(1):17–41. doi: 10.1017/jea.2015.3

[8] Chowdhury SS, Saquib N, Zawad N, Mandal MK, Haque S. Statement Networks: A Power Structure Narrative as Depicted by Newspapers. arXiv. 2018.

[9] Traag VA, Reinanda R, van Klinken G. Elite Co-Occurrence in the Media. Asian Journal of Social Science. 2015;43(5):588–612. doi: 10.1163/15685314-04305005

[10] Mahmood B, Menezes R. United States Congress Relations According to Liberal and Conservative Newspapers. In: 2013 IEEE 2nd Network Science Workshop (NSW). 2013. p. 98–101.

[11] Mahdavi P. Scraping Public Co-Occurrences for Statistical Network Analysis of Political Elites. Political Science Research and Methods. 2019;7(2):385–392. doi: 10.1017/psrm.2017.28

[12] Düring M. Can Network Analysis Reveal Importance? Degree Centrality and Leaders in the EU Integration Process. In: Aiello LM, McFarland D, editors. Social Informatics. Lecture Notes in Computer Science. Cham: Springer International Publishing; 2015. p. 314–318.

[13] Xue K, Zhou Y, Ma Z, Ruan T, Zhang H, He P. Fine-Tuning BERT for Joint Entity and Relation Extraction in Chinese Medical Text. In: IEEE International Conference on Bioinformatics and Biomedicine (BIBM). 2019. p. 892–897.

[14] Wei Z, Su J, Wang Y, Tian Y, Chang Y. A Novel Cascade Binary Tagging Framework for Relational Triple Extraction. In: Proceedings of the 58th Annual Meeting of the Association for Computational Linguistics. 2020. p. 1476–1488.

[15] Wang Y, Yu B, Zhang Y, Liu T, Zhu H, Sun L. TPLinker: Single-stage Joint Extraction of Entities and Relations Through Token Pair Linking. In: Proceedings of the 28th International Conference on Computational Linguistics. 2020. p. 1572–1582.

[16] Zhong Z, Chen D. A Frustratingly Easy Approach for Entity and Relation Extraction. In: Proceedings of the 2021 Conference of the North American Chapter of the Association for Computational Linguistics: Human Language Technologies. 2021. p. 50–61.

[17] Zhao K, Xu H, Cheng Y, Li X, Gao K. Representation Iterative Fusion Based on Heterogeneous Graph Neural Network for Joint Entity and Relation Extraction. Knowledge-Based Systems. 2021;219:106888. doi: 10.1016/j.knosys.2021.106888

[18] Zheng S, Wang F, Bao H, Hao Y, Zhou P, Xu B. Joint Extraction of Entities and Relations Based on a Novel Tagging Scheme. ArXiv. 2017;abs/1706.05075.

[19] Dai D, Xiao X, Lyu Y, Dou S, She Q, Wang H. Joint Extraction of Entities and Overlapping Relations Using Position-Attentive Sequence Labeling. In: AAAI Conference Proceedings. 2019;33:6300–6308.

[20] Simmering PF, Huoviala P. Large Language Models for Aspect-Based Sentiment Analysis. arXiv preprint arXiv:2310.18025. 2023.

[21] Cohen LR, Noll RG. How to Vote, Whether to Vote: Strategies for Voting and Abstaining on Congressional Roll Calls. Political Behavior. 1991;13(2):97–127. doi: 10.1007/BF00992292

[22] Grover A, Leskovec J. node2vec: Scalable Feature Learning for Networks. Proceedings of the 22nd ACM SIGKDD International Conference on Knowledge Discovery and Data Mining. 2016;855–864. 10.1145/2939672.2939754 PMC5108654 27853626

