# Supplementary material for: A frustratingly easy way of extracting political networks from text
Source: PLoS One. 2025 Jan 27;20(1):e0313149. doi: 10.1371/journal.pone.0313149 (PMC11771885; doi:10.1371/journal.pone.0313149)
Supplement: S2 Appendix — (PDF) [file pone.0313149.s002.pdf]

S2 Appendix: Regression table for experiment 1

Naim Bro

	Dependent variable: Legislative agreement	
	(1)	(2)
Link (ref = no link)	0.109***	
	(0.037)	
Negative (ref = neutral)		-0.415***
		(0.082)
Positive (ref = neutral)		-0.060
		(0.105)
Same party	0.386***	0.377***
	(0.037)	(0.111)
Same region	-0.141***	-0.100
	(0.023)	(0.083)
Same sector	1.197***	0.989***
	(0.019)	(0.080)
Constant	-0.314***	-0.014
	(0.009)	(0.058)
Observations	11,921	529
R^2	0.321	0.382
Adjusted R^2	0.321	0.377
Residual Std. Error	0.824 (df = 11916)	0.787 (df = 523)
F Statistic	1411.549*** (df = 4.0; 11916.0)	64.791*** (df = 5.0; 523.0)

Note: The values of the dependent variable are expressed in standard deviations. Standard errors are given in parentheses. *p<0.1; **p<0.05; ***p<0.01
