# Supplementary material for: A frustratingly easy way of extracting political networks from text
Source: PLoS One. 2025 Jan 27;20(1):e0313149. doi: 10.1371/journal.pone.0313149 (PMC11771885; doi:10.1371/journal.pone.0313149)
Supplement: S3 Appendix — (PDF) [file pone.0313149.s003.pdf]

S3 Appendix: Regression table for experiment 2

Naim Bro

	Dependent variable: Legislative agreement	
	(1)	(2)
Cosine distance unweighted std	-0.116***	
	(0.035)	
Cosine distance weighted std		-0.211***
		(0.035)
Same party	0.383***	0.354***
	(0.113)	(0.111)
Same region	-0.082	-0.086
	(0.084)	(0.082)
Same sector	1.009***	0.987***
	(0.081)	(0.079)
Intercept	-0.461***	-0.446***
	(0.049)	(0.048)
Observations	529	529
R^2	0.365	0.395
Adjusted R^2	0.360	0.391
Residual Std. Error	0.800 (df = 524)	0.781 (df = 524)
F Statistic	75.328*** (df = 4.0; 524.0)	85.643*** (df = 4.0; 524.0)

Note: The values of the dependent variable are expressed in standard deviations. Cosine distance (unweighted and weighted) is also expressed in standard deviations. Standard errors are given in parentheses. *p<0.1; **p<0.05; ***p<0.01
