# Supplementary material for: A frustratingly easy way of extracting political networks from text
Source: PLoS One. 2025 Jan 27;20(1):e0313149. doi: 10.1371/journal.pone.0313149 (PMC11771885; doi:10.1371/journal.pone.0313149)
Supplement: S4 Appendix — (PDF) [file pone.0313149.s004.pdf]

S4 Appendix: Network descriptive statistics

Naim Bro

	Value
Number of Nodes	151
Number of Edges	529
Density	0.0467
Average Degree	7.007
Average Clustering Coefficient	0.240
Average Path Length	2.842
Number of Connected Components	1
