# Supplementary material for: A frustratingly easy way of extracting political networks from text
Source: PLoS One. 2025 Jan 27;20(1):e0313149. doi: 10.1371/journal.pone.0313149 (PMC11771885; doi:10.1371/journal.pone.0313149)
Supplement: S5 Appendix — (PDF) [file pone.0313149.s005.pdf]

S5 Appendix: Experiments 1 and 2 with GPT-4o-mini

Naim Bro

Experiment 1

	Dependent variable: Legislative agreement	
	(1)	(2)
Link (ref = no link)	-0.029	
	(0.037)	
Negative (ref = neutral)		-0.232***
		(0.084)
Positive (ref = neutral)		0.074
		(0.099)
Same party	0.394***	0.430***
	(0.037)	(0.102)
Same region	-0.136***	-0.070
	(0.023)	(0.083)
Same sector	1.201***	1.037***
	(0.019)	(0.084)
Constant	-0.310***	-0.240***
	(0.009)	(0.060)
Observations	11,933	550
R^2	0.321	0.399
Adjusted R^2	0.321	0.394
Residual Std. Error	0.824 (df = 11,928)	0.810 (df = 544)
F Statistic	1410.000*** (df = 4; 11,928)	72.380*** (df = 5; 544)
Note:		*p<0.1; **p<0.05; ***p<0.01

Experiment 2

	Dependent variable: Legislative agreement	
	(1)	(2)
Unweighted Cosine Distance (std)	-0.163***	
	(0.034)	
Weighted Cosine Distance (std)		-0.193***
		(0.034)
Same party	0.402***	0.359***
	(0.097)	(0.097)
Same region	-0.045	-0.045
	(0.079)	(0.079)
Same sector	0.950***	0.953***
	(0.080)	(0.079)
Constant	-0.514***	-0.507***
	(0.048)	(0.048)
Observations	550	550
R^2	0.413	0.423
Adjusted R^2	0.409	0.418
Residual Std. Error	0.769 (df = 545)	0.763 (df = 545)
F Statistic	96.049*** (df = 4; 545)	99.777*** (df = 4; 545)

Note: *p<0.1; **p<0.05; ***p<0.01
