# Supplementary material for: A frustratingly easy way of extracting political networks from text
Source: PLoS One. 2025 Jan 27;20(1):e0313149. doi: 10.1371/journal.pone.0313149 (PMC11771885; doi:10.1371/journal.pone.0313149)
Supplement: S6 Appendix — (PDF) [file pone.0313149.s006.pdf]

S6 Appendix: News Clip Example and Resulting Structured Dictionary

Naim Bro

The following example illustrates how this methodology was applied to a sample news clip (translated from Spanish to English):

> ”The rejection of the Tax Reform in Congress last Wednesday represented a setback for La Moneda. The vote ended with recriminations in the Chamber of Deputies, as only 2 votes were missing to approve the idea of legislating. There were some absences that drew attention, such as the case of Pamela Jiles, whose support was reportedly already secured for the government. For this reason, Deputy Karol Cariola decided to confront her colleague, shouting ’you screwed over Chile’ amid ironic applause. The absence of Viviana Delgado, a deputy who had previously accused Minister Ávila of verbal aggression, was also noted.”

Processed Output (JSON Format):


```
{
  "nodes": [
    { "id": 1, "label": "Pamela Jiles", "type": "person" },
    { "id": 2, "label": "Karol Cariola", "type": "person" },
    { "id": 3, "label": "Viviana Delgado", "type": "person" },
    { "id": 4, "label": "Ministro Ávila", "type": "person" }
  ],
  "edges": [
    {
      "from": 2,
      "to": 1,
      "label": "criticized",
      "sentiment": -0.8
    },
    {
      "from": 2,
      "to": 1,
      "label": "shouted at",
      "sentiment": -0.9
    },
    {
      "from": 3,
      "to": 4,
      "label": "accused of verbal aggression",
      "sentiment": -0.7
    }
  ]
}
```


This example demonstrates how the system processed the raw text to extract key political figures and their relationships, with sentiment scores assigned to characterize the nature of their interactions. In this case, interactions between Karol Cariola and Pamela Jiles were assigned high negative sentiment scores,

while Viviana Delgado's accusation against Ministro Ávila was also categorized with a moderately negative score.
